# Ultrasound and Radiological Patterns in Assessing the Efficacy of Juvelook for Collagen Stimulation and Tear Trough Enhancement: A Case Report

**DOI:** 10.7759/cureus.82668

**Published:** 2025-04-21

**Authors:** Karina Ravera

**Affiliations:** 1 Radiology, Sanatorio Trinidad Mitre, Buenos Aires, ARG

**Keywords:** cannula injection, hyaluronic acid (ha), juvelook, poly-l-lactic acid (pdlla), tear trough rejuvenation

## Abstract

Tear trough deformity, in the presence of hollows, shadows, and lax skin underneath the eyes, is a common cosmetic concern. Frequent application of non-surgical treatments, such as hyaluronic acid (HA) fillers and fat grafting, can result in complications, such as the Tyndall effect and vascular occlusion. Juvelook (VAIM Global Inc., Seoul, South Korea), a new hybrid filler HA/poly-D,L-lactic acid (PDLLA), exhibits immediate volumization through gain in skin thickness and secondary delayed collagen stimulation.

A 24-year-old female with moderate hollowness, pigmentation, and laxity of the skin in the under-eye region required non-surgical rejuvenation. Clinical and radiological evaluations (ultrasonography, CT, MRI) were conducted. Bilateral Juvelook injection was performed using a supraperiosteal and subdermal cannula. On day 18 after the procedure, the patient demonstrated a better skin texture, reduced hollowness, and very high satisfaction. Imaging revealed excellent integration of the filler with no side effects.

Juvelook can potentially serve as an effective and safe solution for tear trough rejuvenation. HA injects immediate volumization, whereas PDLLA stimulates collagen to ensure long-lasting correction. The cannula technique keeps complications at bay in such delicate areas. The absence of adverse effects and satisfactory results ensures biocompatibility and effectiveness. Large controlled trials, however, need to be performed to confirm these findings and compare them to existing treatments.

This case report documents the efficacy of Juvelook in tear trough rejuvenation with immediate volumetric gain and skin texture enhancement. The cannula technique minimized risk, and imaging confirmed its biocompatibility. Therefore, additional studies are required to establish long-term safety, optimal injection techniques, and comparisons with other treatment modalities.

## Introduction

The tear trough, located below the lower eyelid, is a common aesthetic concern, particularly with aging, which results in thinner skin, loss of subcutaneous tissue, and structural orbital/malar changes. Dark circles, hollows, fine lines, and skin laxity often affect self-esteem and social confidence [1,2].

Hyaluronic acid (HA) fillers, fat grafting, and topical treatments are commonly used non-surgical methods to improve the appearance of the under-eye area, known as the "tear trough." Although these treatments are popular, they can sometimes lead to unwanted side effects. For instance, the Tyndall effect can cause bluish discoloration under the skin when HA fillers are used improperly. Additionally, there are risks such as edema (swelling) and vascular occlusion (blocking of blood vessels), which are particularly concerning given the sensitive nature of the eye area [3,4]. Because of these potential issues, researchers have been motivated to develop safer alternatives. One such innovative solution is Juvelook (VAIM Global Inc., Seoul, South Korea), a hybrid filler that combines poly-D,L-lactic acid (PDLLA) with noncross-linked HA. This unique formulation allows Juvelook to merge naturally with the body's skin tissue. It slowly integrates into the dermal matrix of the skin, which is a densely packed structural layer beneath the surface. This slow integration helps prevent common problems such as lumps or an unnatural look, enhancing the skin's texture and moisture levels in a way that appears smooth and natural. Moreover, Juvelook acts on both the deeper and surface layers of the skin. This dual action makes it particularly effective for rejuvenating the tear trough area, providing a refreshed look while minimizing the risks associated with older treatments [5-8]. This makes Juvelook a promising option for those seeking safe and effective nonsurgical eye rejuvenation.

Despite their potential, the efficacy of PDLLA and HA has been disputed. While some studies, such as Signori et al. (2024), confirm PDLLA's effects on collagen stimulation, others, such as Haddad et al. (2024), are unconvinced by its effectiveness. Similarly, Liu et al. (2024) found HA to be effective, in contrast to the findings of Anido et al. (2023) [3,9-11].

This case report determines the safety and efficacy of Juvelook, a hybrid filler combining PDLLA and HA, in non-surgical tear trough rejuvenation in a 24-year-old female, highlighting its biostimulative property and patient satisfaction.

## Case presentation

**Figure 1:**
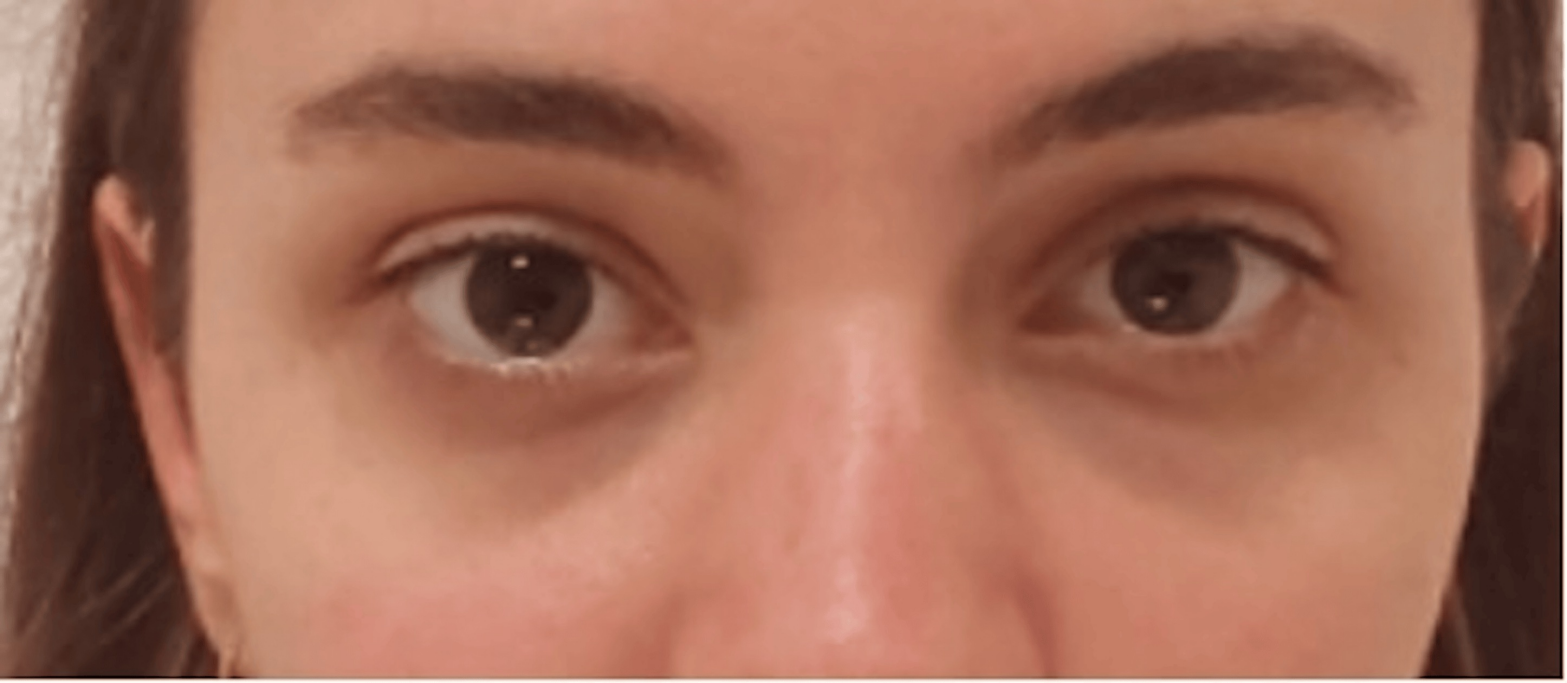
Front- before the treatment.

A 24-year-old female presented with moderate hollowness, pigmentation, and mild skin laxity in the tear trough area, which created a fatigued appearance (Figure 1). She had no prior medical illnesses, drug allergies, or cosmetic treatment in the periorbital region. The patient expressed low self-confidence as a result of her appearance, especially in her 20s, desiring a non-surgical, gradual, and organic option for rejuvenation. Her aim was to restore a youthful and revitalized appearance through noninvasive means.

**Figure 2:**
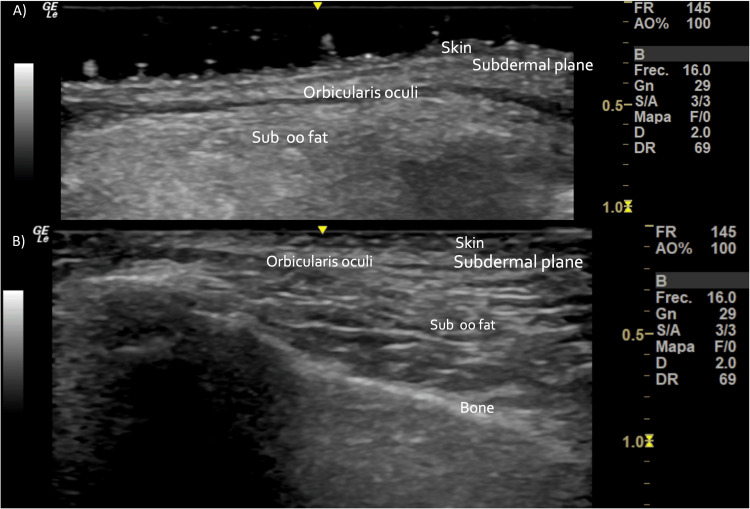
Skin and soft tissue high-resolution ultrasound before treatment (A), immediately after treatment (B). A slight increase in tissue echogenicity is visualized in all the tissue planes.

Clinical evaluation revealed severe bilateral tear trough deformity consisting of accentuated nasojugal grooves and shadows, which were intensified by facial movement. Mild skin elasticity was lost, but there were no signs of acute skin or vascular disease. Baseline investigations like ultrasound (Figure 2), CT, and MRI revealed no underlying abnormality nor evidence of any residual filler, qualifying the patients for biostimulator-filler treatment. Informed consent was obtained from the patient after properly detailing the possible complications, benefits, and postoperative expectations. 

Juvelook, a HA/PDLLA hybrid injectable, was chosen for its immediate volumizing effect and collagen stimulation over time. One vial of Juvelook (PDLLA plus HA, bidistilled water-9cc, lido-1cc) was injected bilaterally with a 27G cannula. Considering the supraperiosteal plane, 4cc was injected, whereas in the subdermal plane, 5cc was injected. Improvement in tear trough area was achieved in only one session. 

**Figure 3:**
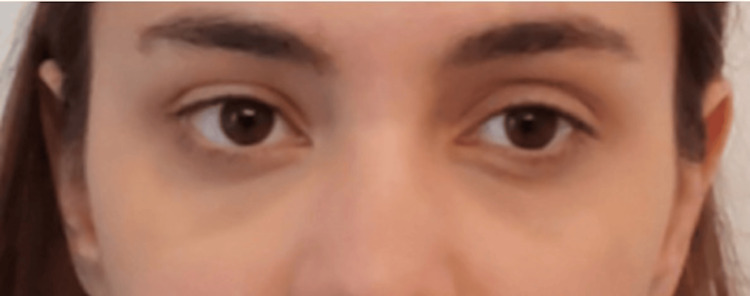
Digital front photograph at two weeks after juvelook treatment.

At the 18-day follow-up, the patient noted improvement in skin texture, decreased hollowing of the tear trough, and an increased reduction of shading in the under-eye area (Figure 3). Imaging evaluation demonstrated a mild augmentation of echogenicity in this area, suggesting good tissue integration of the filler material. CT and MRI results showed no alteration in tissue density or radiological patterns, indicating good biocompatibility and safety of juvelook.

**Figure 4:**
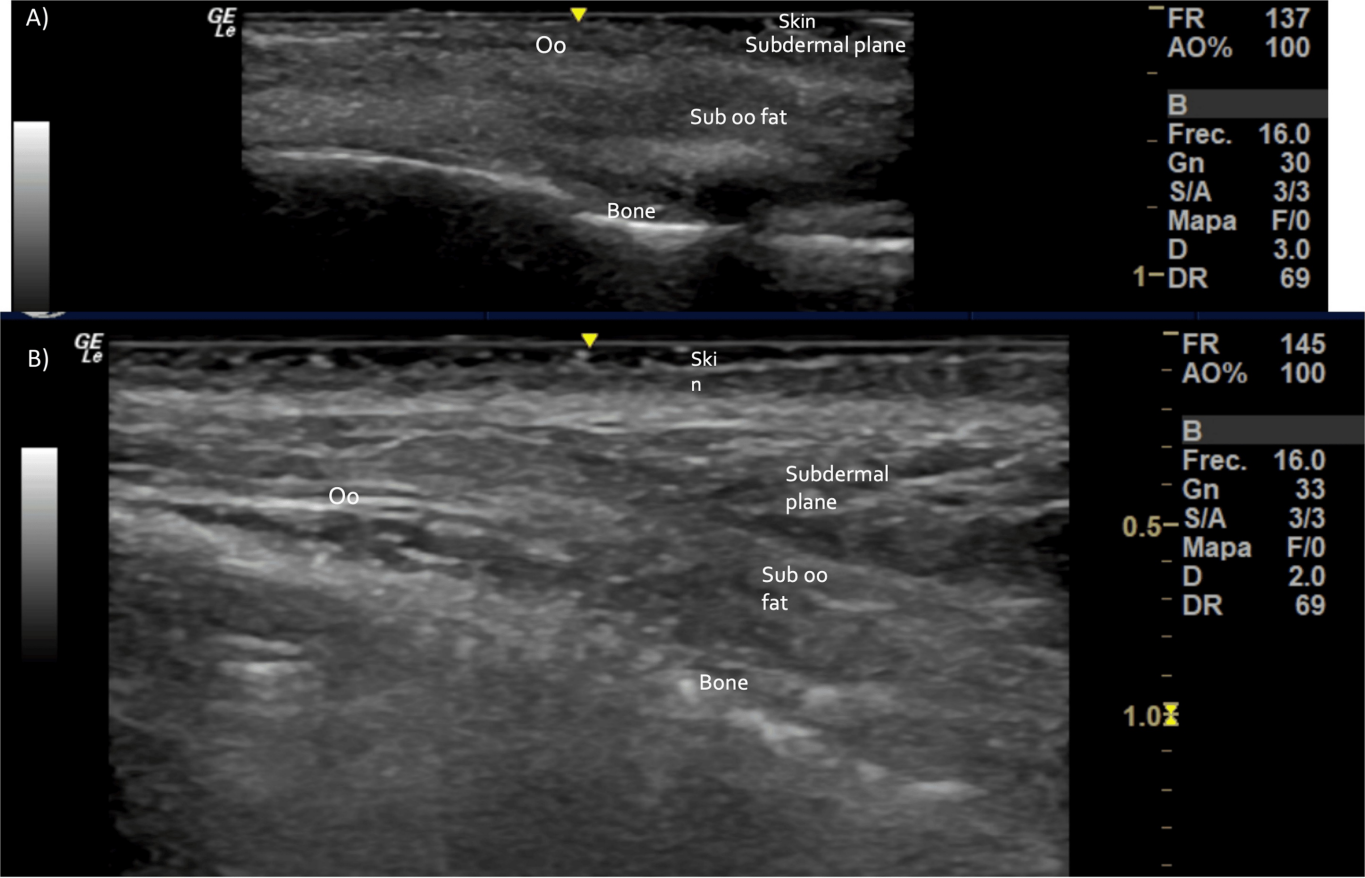
Skin and soft tissue high-resolution ultrasound, immediately after treatment: a slight increase in tissue echogenicity is visualized in all the tissue planes. Images are shown using the medial-view (A) and lateral-view (B).

**Figure 5:**
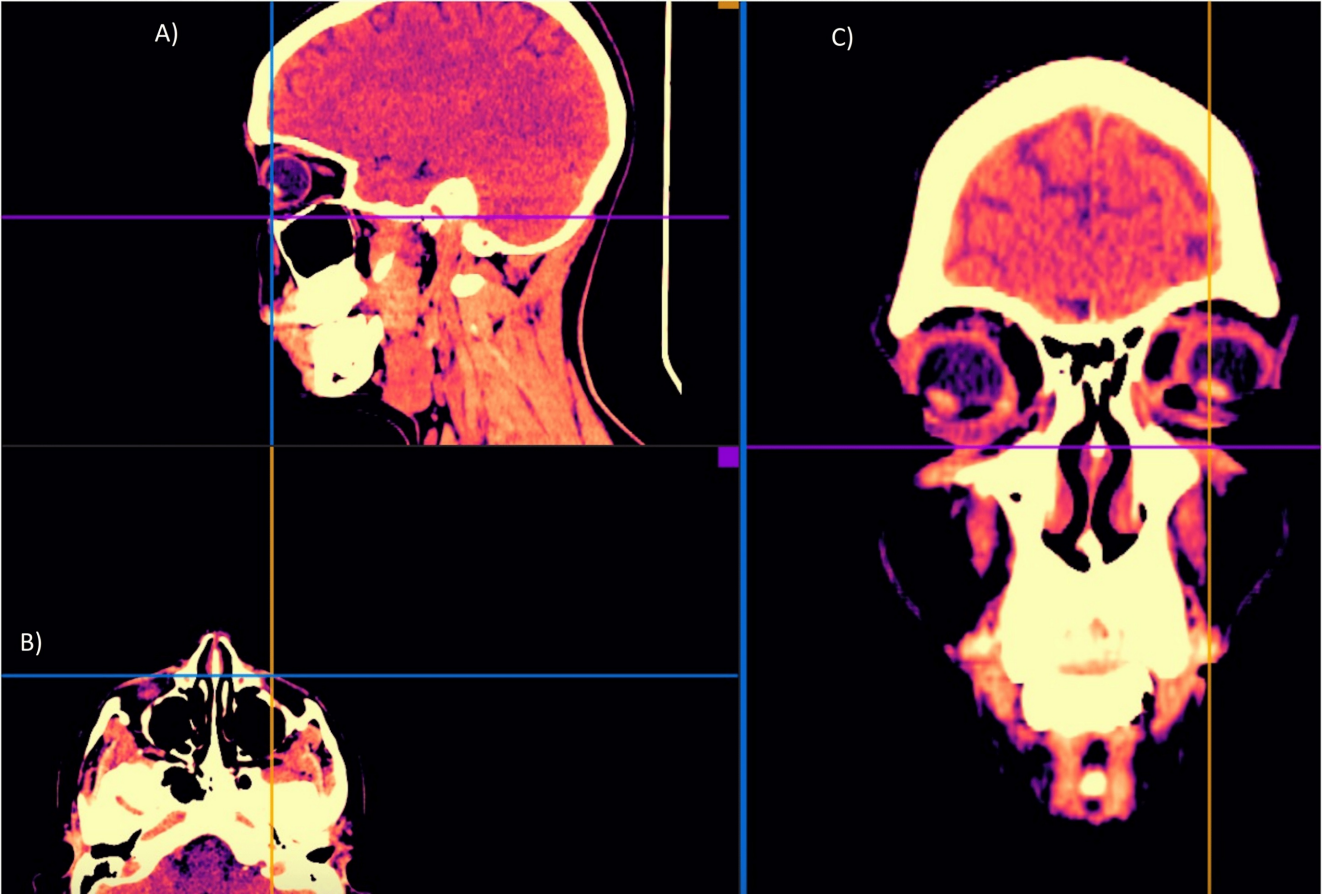
Facial CT multiplanar reconstruction (MPR) right lacrimal sulcus or multiplanar reconstruction. In these images it was possible to demonstrate in the three planes of space through a sagittal (A), coronal (B) and axial (C) views that Juvelook did not alter the tissue density or radiological CT pattern of the tissues after having been applied (two weeks post injection).

**Figure 6:**
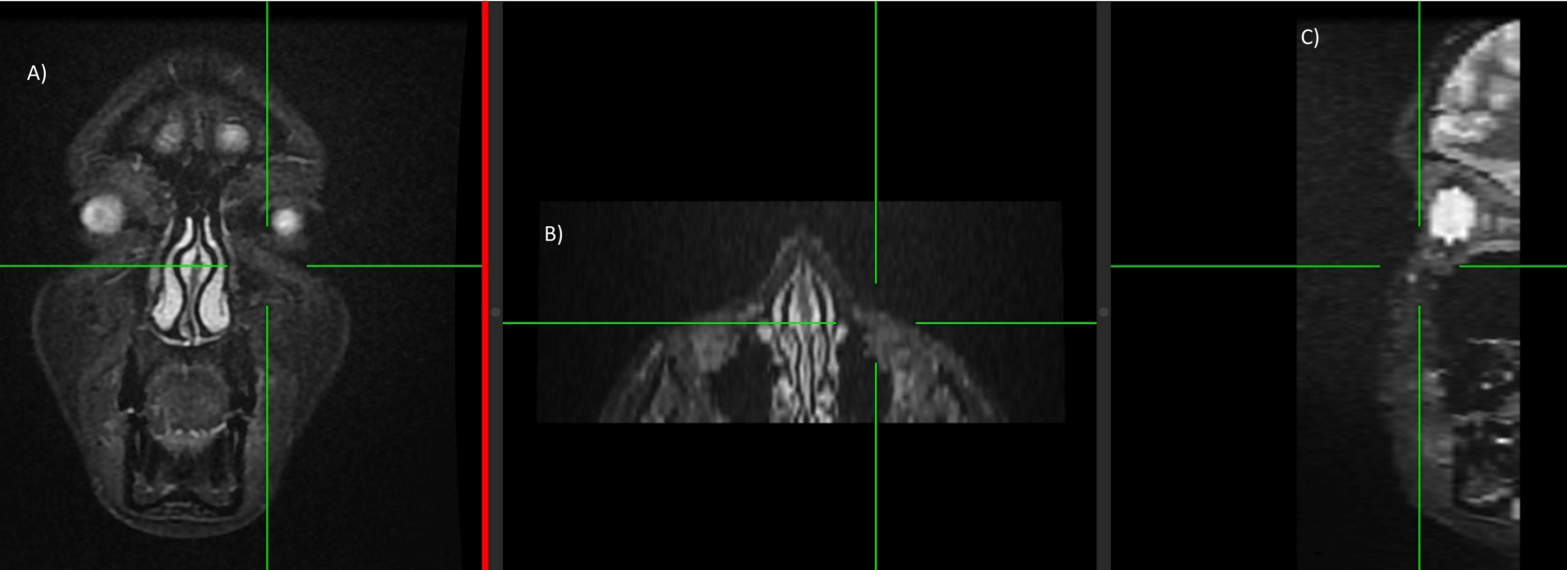
Facial MRI maximum intensity projection (MIP); these images are able to demonstrate in the three spatial planes through a coronal (A), axial (B) and sagittal (C) view in MRI that Juvelook did not alter the signal intensity or radiological pattern in the MRI of the tissues after having been applied (two weeks post injection).

**Figure 7:**
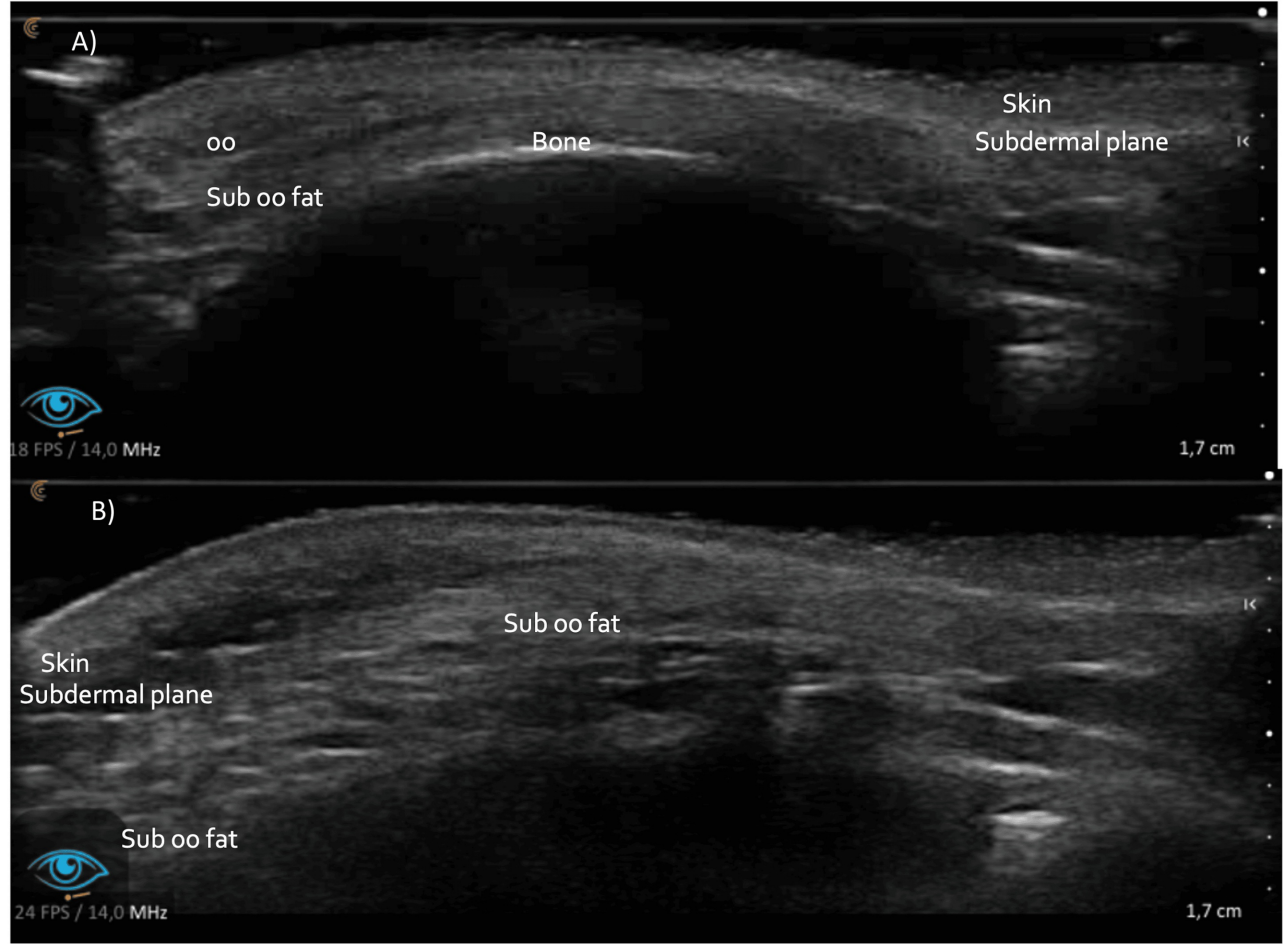
Skin and soft tissue high-resolution ultrasound two weeks post-treatment: Normal soft tissue ultrasound patterns. Images are shown using the medial-view (A) and lateral-view (B).

Both practitioners and patient reported 10/10 maximum satisfaction. Imaging studies, including facial magnetic resonance imaging (MRI), computed tomography (CT), and ultrasound (US), were performed to evaluate radiological patterns and soft tissue changes after treatment (Figures 4-7).

High-resolution ultrasound scans using 15-, 18-, and 20-MHz linear probes were performed pre-, post-, and two weeks after PDLLA plus HA treatment (Figures 2, 4, 7). These include transverse and longitudinal sections of the lacrimal sulcus areas. No effect on ultrasound transmission was observed because of the biostimulatory material. Immediately after treatment, mild enhancement of echogenicity in the tear trough tissue planes was noted. No changes in ultrasonographic anatomy were detected after 18 days.

**Figure 8:**
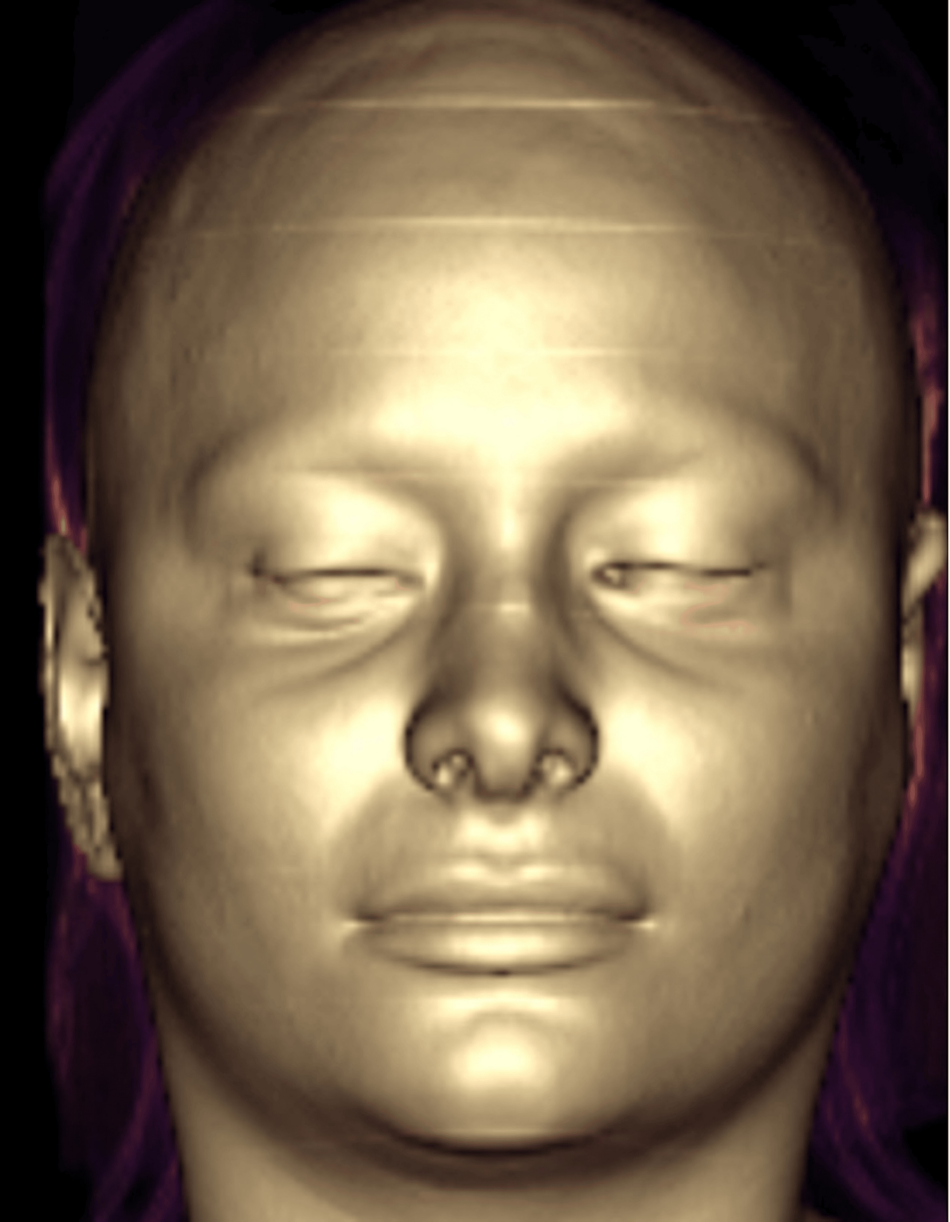
Facial CT: 3D reconstruction skin surface rendering.

**Figure 9:**
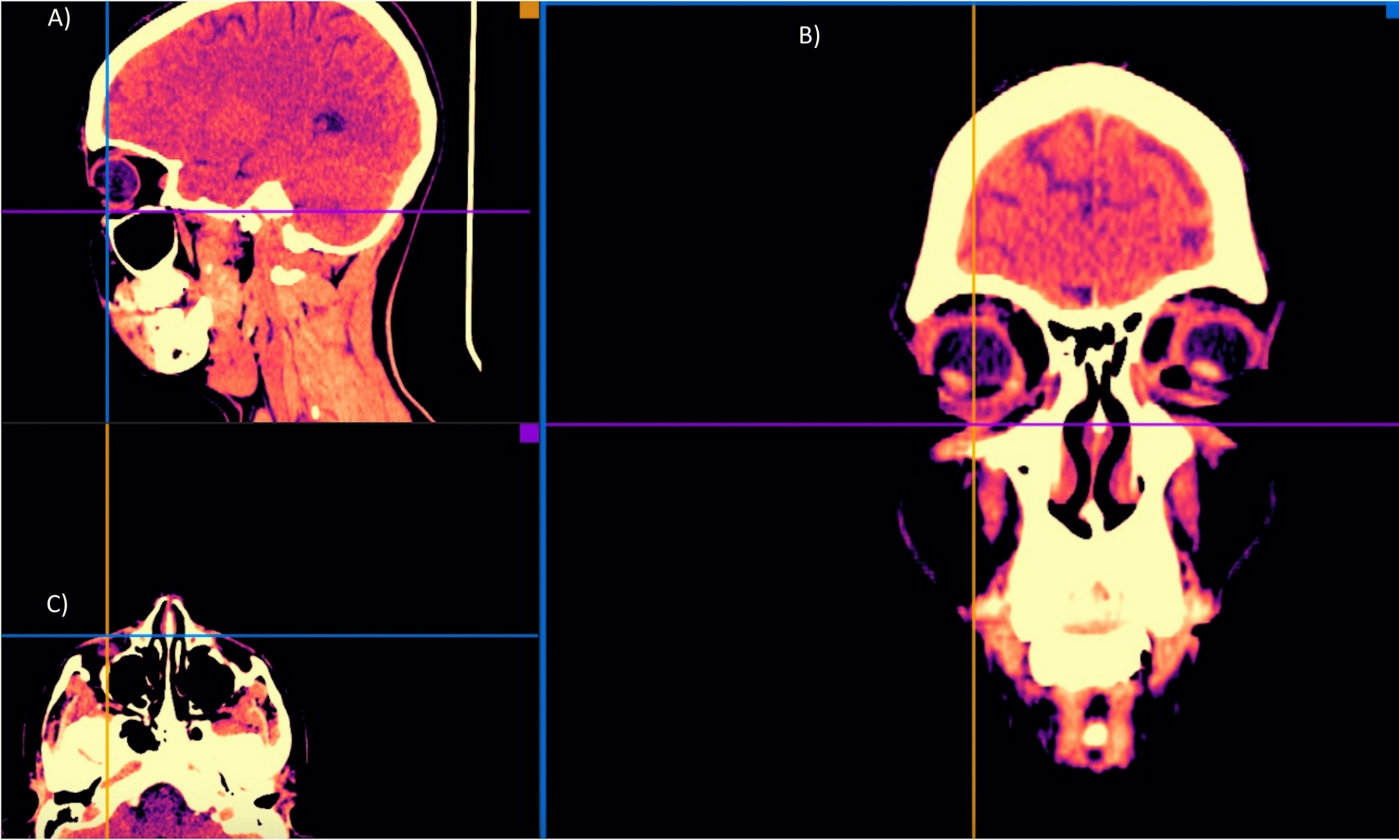
Facial CT multiplanar reconstruction (MPR) left lacrimal sulcus or multiplanar reconstruction. In these images it was possible to demonstrate in the three planes of space through a sagittal (A), coronal (B) and axial (C) view that Juvelook did not alter the tissue density or radiological CT pattern of the tissues after having been applied (two weeks post injection).

**Figure 10:**
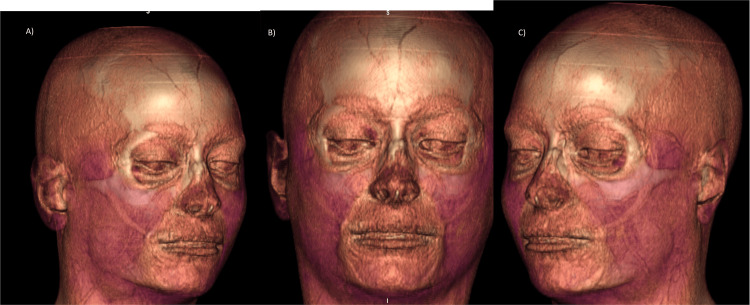
Facial CT 3D reconstruction after the treatment. In these three-dimensional MR images it was possible to demonstrate that Juvelook did not alter the signal intensity or radiological pattern of the tissues after it was applied (two weeks post injection). Views: (A) Right lateral (B) Front (C) Left lateral.

Facial multi-slice CT scans (Figures 5, 8-10) were performed using General Electric Medical Systems equipment (BrightSpeed; Chicago, IL, USA). The left and right lacrimal sulcus were imaged in-slice with a thickness of 1.25 mm, parameters of 120.0 kV, 180.0 mA, and 0.8 s exposure.

**Figure 11:**
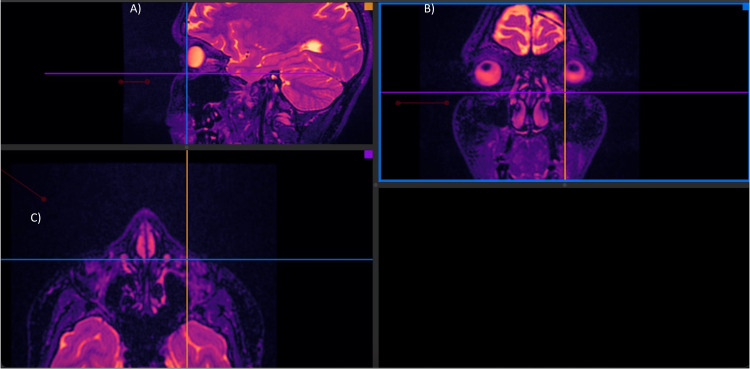
Facial MRI multiplanar reconstruction (MPR). These images can demonstrate in the three planes of space through a sagittal (A), coronal (B) and (C) axial view that Juvelook did not alter the signal intensity or radiological pattern of the MRI of the tissues after having been applied (two weeks post injection).

**Figure 12:**
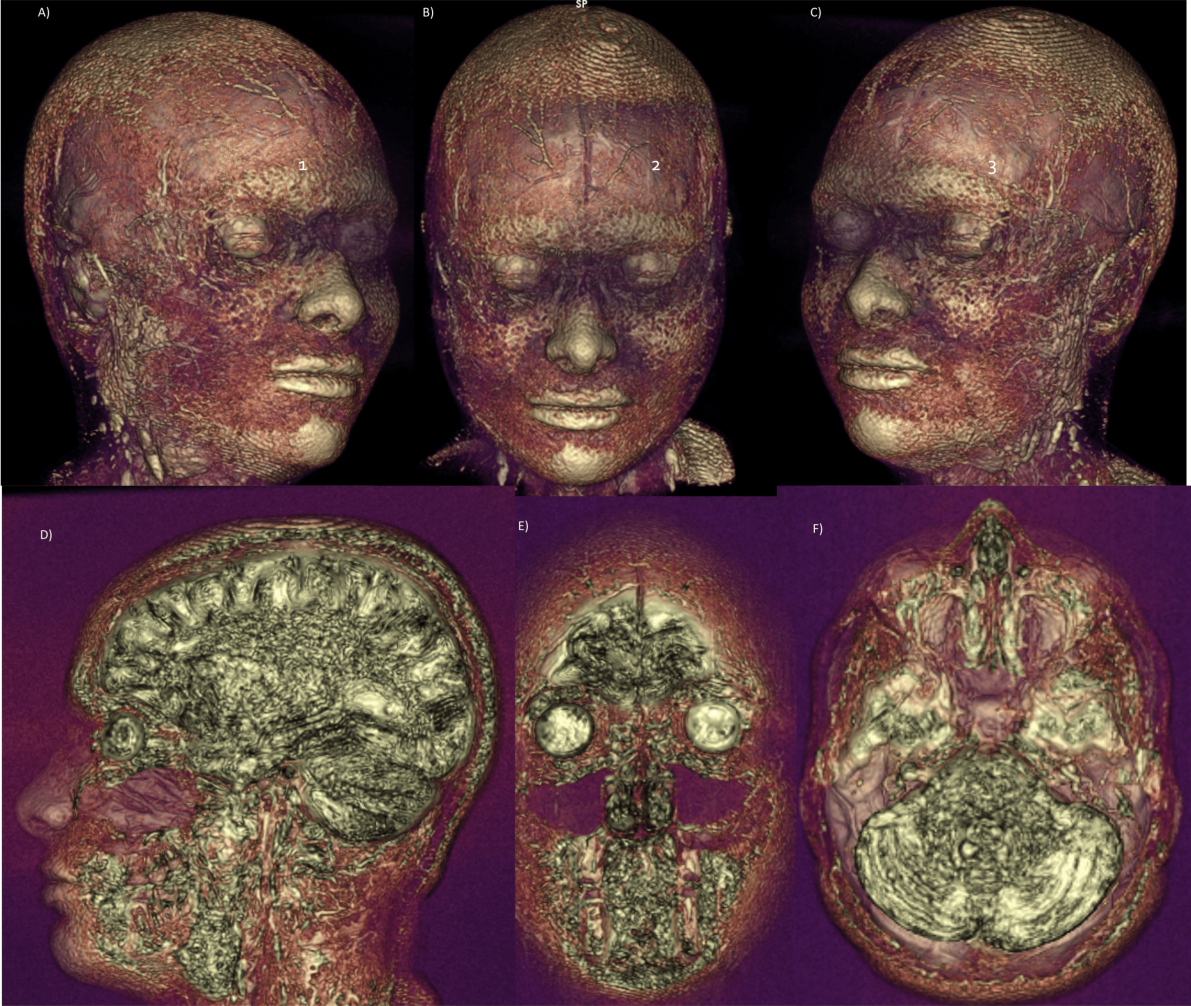
Facial MRI 3D reconstructions, in the above three images: In these three-dimensional MR images it was possible to demonstrate that Juvelook did not alter the signal intensity or radiological pattern of the tissues after it was applied (two weeks post injection). Views: (A) Right lateral, (B) Front, (C) Left lateral. And in the below three, sagittal (D), coronal (E) and axial (F) slices: no alteration of the signal intensity normally visualized in soft tissues after Juvelook injection in the lacrimal sulcus (two weeks post injection).

Facial MRI (Figures 6, 11-12) was performed in a 1.5 Tesla closed resonator with a dedicated facial antenna. Multiplanar slices (sagittal, axial, and coronal) were acquired using weighted sequences that could detect tissue fillers without modifying their normal radiological patterns.

Soft tissue changes and side effects were not observed in any of the imaging studies. At the end of the follow-up period, the patient denied any adverse reactions such as swelling, erythema, or nodularity. The patient expressed high satisfaction with the natural and youthful appearance imparted by treatment.

## Discussion

This case report accurately described the success of Juvelook, an HA/PDLLA hybrid filler, in tear trough rejuvenation in a young female patient. The patient presented with moderate under-eye hollowness, pigmentation, and mild skin laxity, which imparted adjacent tiredness. For nonsurgical interventions in the periorbital region, a more conservative approach is preferred owing to the subtle anatomy of this region [12]. Although effective, conventional HA fillers may present with complications including the Tyndall effect, swelling, and vascular occlusion [13]. Moreover, some HA fillers require frequent injections to maintain their effect, while others can lead to long-term clinical and radiological problems. 

Juvelook utilizes dual mechanisms for immediate volumization, owing to its HA load, and for long-term collagen stimulation via PDLLA. This results in gradual and longer-lasting improvements in skin texture and volume [14]. Juvelook deals with both immediate volume loss and tissue regeneration over time. The skin texture improvement gained by the patient was accompanied by decreased hollowness and reduced shadowing with high patient satisfaction.

The use of cannula-injection techniques also minimizes complications. Cannulas, unlike sharp needles, reduce the risk of vascular injury and bruising [15]. This is particularly relevant in the periorbital region, which is nourished by an intricate vascular network.

Sophisticated imaging techniques are essential for assessing the compatibility and safety of Juvelook. An increase in soft tissue echogenicity was noted after the procedure, which is likely representative of the PDLLA microparticles present within the tissue. This hyperechogenicity may indicate the presence of filler integration. Diffusion of hyperechogenicity over extracellular matrix components may persist during the two-week assessment. High-resolution ultrasound at frequencies of 15 MHz, 18 MHz, and 20 MHz provides this information and has become indispensable for real-time imaging of filler placement, tissue integration, and complication detection, such as vascular compromise or filler misplacement, in facial aesthetic medicine [16]. Computed tomography (CT) and magnetic resonance imaging (MRI) provided further evidence against adverse tissue reactions with ultrathin slices (1.25 mm for CT and 1.2 mm for MRI), yielding detailed anatomical information. These imaging methods provide an objective assessment of the safety and efficacy of aesthetic interventions [17,18].

The findings of this case report are in agreement with those of previous studies of PDLLA and HA fillers for the rejuvenation of tear troughs. For example, Signori et al. demonstrated that PLLA activates collagen production and improves skin texture, thereby proving the long-term benefits noted here [9]. Rohrich et al. also emphasized the immediate volumizing and hydrating effects of HA that correlated with the immediate improvement experienced by the patient [19]. Such diverging reports, like those of Lowe et al. (2005), suggest that the effectiveness of PLLA in rejuvenating skin may be variable and call for further studies on such effects in combination with hybrid fillers such as Juvelook [20]. This comparison reiterates the personalized approach to treatment concerning the need for larger controlled studies to endorse the efficacy and safety of Juvelook as compared to other treatments.

The limitations of this case report highlight several areas requiring further investigation. First, the study involved only a single patient, which, while yielding promising results, underscores the need for larger and more controlled studies to establish the efficacy and safety of Juvelook more reliably. Such studies would also facilitate comparisons with other treatments and help optimize injection techniques to sustain long-term results. Additionally, the follow-up period in this study was only 18 days, which is insufficient to fully evaluate long-term collagen stimulation, which is a critical benefit claimed for PDLLA-based fillers such as Juvelook. Another limitation was the absence of a comparative group, such as patients treated with standard HA fillers, which would provide a benchmark for assessing Juvelook's relative performance. Finally, while the imaging results are thoroughly documented, the study lacks histological confirmation, such as collagen biopsy, which would provide deeper insights into the mechanisms underlying the observed effects.

## Conclusions

This case report illustrates the safety and effectiveness of Juvelook, an HA/PDLLA hybrid injectable. Using Juvelook, the patient achieved notable improvement without adverse events due to hollowness, pigmentation, and mild skin laxity. This has further reduced the risk through the application of a cannula.

Radiological follow-up was performed using high-resolution ultrasound probes (15, 18, and 20 MHz), multislice CT (1.25 mm slices with 3D post-processing), and high-field MRI (1.2 mm ultra-thin slices with 3D reconstructions) to prove the direct biocompatibility of Juvelook and to exclude any tissue reaction. Advanced imaging techniques have revealed a detailed anatomy that supports the safety and efficacy of the product. However, it should be noted that this is a finding in only one case, and further controlled studies should be conducted. Further studies are needed to determine the long-term end results, best injection techniques, and comparative effectiveness with existing treatments before Juvelook can be launched as a new alternative to tear trough rejuvenation.
